# Short-term, but not acute, intake of New Zealand blackcurrant extract improves insulin sensitivity and free-living postprandial glucose excursions in individuals with overweight or obesity

**DOI:** 10.1007/s00394-020-02329-7

**Published:** 2020-07-09

**Authors:** A. Nolan, R. Brett, J. A. Strauss, C. E. Stewart, S. O. Shepherd

**Affiliations:** Research Institute for Sport and Exercise Sciences, Liverpool John Moores University, Liverpool, UK

**Keywords:** Anthocyanin, Insulin resistance, Polyphenol, Obesity

## Abstract

**Abstract:**

Impaired postprandial glucose handling and low-grade systemic inflammation are risk factors for developing insulin resistance in individuals with overweight or obesity. Acute ingestion of anthocyanins improves postprandial glucose responses to a single carbohydrate-rich meal under strictly controlled conditions.

**Purpose:**

Examine whether acute and short-term supplementation with anthocyanin-rich New Zealand blackcurrant (NZBC) extract can improve postprandial glucose responses to mixed-macronutrient meals.

**Methods:**

Twenty-five overweight (BMI > 25 kg m^2^) sedentary individuals participated in one of the following double-blinded, randomised controlled trials: (1) ingestion of 600 mg NZBC extract or placebo prior to consumption of a high-carbohydrate, high-fat liquid meal (*n* = 12); (2) 8-days supplementation with NZBC extract (600 mg day^−1^) or placebo, with insulin sensitivity and markers of inflammation assessed on day-7, and free-living postprandial glucose (continuous glucose monitoring) assessed on day-8 (*n* = 13).

**Results:**

A single dose of NZBC extract had no effect on 3 h postprandial glucose, insulin or triglyceride responses. However, in response to short-term NZBC extract supplementation insulin sensitivity was improved (+ 22%; *P* = 0.011), circulating C-reactive protein concentrations decreased (*P* = 0.008), and free-living postprandial glucose responses to both breakfast and lunch meals were reduced (− 9% and − 8%, respectively; *P* < 0.05), compared to placebo.

**Conclusion:**

These novel results indicate that repeated intake, rather than a single dose of NZBC extract, is required to induce beneficial effects on insulin sensitivity and postprandial glucose handling in individuals with overweight or obesity. Continuous glucose monitoring enabled an effect of NZBC extract to be observed under free-living conditions and highlights the potential of anthocyanin-rich supplements as a viable strategy to reduce insulin resistance.

## Introduction

Recent estimates suggest that nearly two-thirds of adults in the UK are classified as overweight or obese [1]. Individuals who are overweight or obese may exhibit elevated postprandial glucose and triglyceride responses [2], as well as systemic inflammation [3], leading to insulin resistance and an increased risk of type 2 diabetes (T2D) and cardiovascular disease. Emerging evidence has highlighted a potential role for foods and beverages containing flavonoids to reduce T2D risk. In this context, the anthocyanins (a major flavonoid subclass) are of particular interest because prospective studies demonstrate that higher anthocyanin intakes are associated with a lower risk of T2D and cardiovascular disease [4–6]. Several randomised controlled trials also report an ability for foods and beverages rich in anthocyanins to blunt postprandial glucose responses and improve insulin sensitivity [7–14]. Although promising, the currently-available evidence is predominantly limited to single-meal experiments conducted under strictly controlled laboratory conditions [7–9, 12–14], in healthy individuals [7, 8, 12–15], using unsustainable portion sizes [16]. Thus, to truly examine whether anthocyanins can reduce T2D risk, it is vital to explore the ability of anthocyanins to modulate free-living postprandial responses to multiple daily meals with mixed macronutrient contents, and to improve insulin sensitivity in less healthy individuals.

Blackcurrant (*Ribes nigrum*) is one of the richest sources of anthocyanins [17] and it was recently shown that acute ingestion of a blackcurrant extract prior to consumption of a carbohydrate-rich meal reduced postprandial glucose and insulin excursions in healthy individuals [7]. However, whether the acute improvements in postprandial glucose and insulin responses remain when protein and/or fat is included in the test meal is not known. This is important, because dietary fat creates a natural delay in the digestion and absorption of carbohydrate from the gut [18]. Moreover, inclusion of fat in the test meal also allows for the quantification of the postprandial triglyceride response, which is an important factor to consider given that postprandial triglyceride handling is directly related to cardiovascular disease risk [19, 20]. The translation of results obtained using gold standard laboratory approaches to assess postprandial glycaemic responses, such as plasma glucose concentrations following a glucose challenge, are also limited, since they provide no insight into the postprandial response to multiple mixed-macronutrient meals throughout a typical day under ‘real world’ free-living conditions. This can be overcome through the use of continuous glucose monitoring systems (CGMS), which have been used successfully to assess the severity of postprandial hyperglycaemia in T2D patients [21] and in our own lab to investigate the prevalence of hyper- and hypoglycaemic episodes following exercise in people with type 1 diabetes [22]. No previous studies though have investigated the effect of (blackcurrant) anthocyanins on postprandial glucose excursions under free-living conditions.

Nutritional intervention studies investigating the effect of acute consumption of blackcurrant extract on postprandial responses do not provide information regarding the potential adaptation to repeated intake. To try and address this, Willems et al. [15] investigated the effect of short-term (7 days) supplementation with New Zealand blackcurrant (NZBC) powder, and demonstrated reduced postprandial glucose and insulin excursions to a glucose challenge. Whether NZBC powder improved whole-body insulin sensitivity was not assessed. Further, this study did not include a placebo arm and was conducted in healthy individuals. Therefore, a randomised controlled trial is now required to investigate the effects of short-term blackcurrant extract supplementation in individuals with lower insulin sensitivity, such as those with overweight or obesity.

Two separate but inter-related randomised controlled studies were conducted to explore the effectiveness of blackcurrant anthocyanins as a simple nutritional strategy to reduce T2D risk factors in overweight and obese individuals. First, the hypothesis that acute NZBC extract intake would improve postprandial plasma glucose, insulin and triglyceride responses to a high-carbohydrate, high-fat meal was examined. Second, the hypothesis that short-term (8 day) intake of NZBC extract would improve insulin sensitivity and free-living postprandial glucose excursions (under standardised dietary conditions), as well as reduce biomarkers of inflammation in overweight/obese individuals was investigated.

## Methods

### Participants

A total of 25 overweight (BMI 28.8 ± 3.9 kg m^−2^), inactive office-workers to take part in two inter-related but separate studies. Characteristics for participants in each study are presented in Table 1. Participants were deemed to be inactive if they undertook < 1 h structured physical activity per week (in the preceding 6 months). All participants were absent of any other metabolic comorbidities and cardiovascular disease. Both trials were approved by the Liverpool John Moores University Research Ethics Committee. Written, informed consent was obtained following an explanation of the experimental procedures.

**Table 1:** Participant characteristics

	Study 1 (*n* = 12)	Study 2 (*n* = 13)
M/F	6/6	10/3
Age (years)	28 ± 9	30 ± 10
Height (m)	1.73 ± 0.10	1.75 ± 0.10
Body mass (kg)	88.9 ± 16.1	83.6 ± 6.4
BMI (kg m^−2^)	29.9 ± 4.8	27.6 ± 3.3
Lean mass (kg)	62.1 ± 14.2	64.0 ± 7.2
Fat mass (kg)	25.5 ± 12.8	25.5 ± 5.6
Body fat (%)	28.6 ± 4.8	30.5 ± 3.1
Habitual anthocyanin intake (mg day^−1^)	30 ± 25	15 ± 13

Values are means ± S.D

*BMI* body mass index

### Screening procedures

Screening procedures were identical for both studies. During an initial visit, height and weight were measured to determine BMI, and an assessment of body composition was conducted using bioelectrical impedance (Tanita BC 418 MA Segmental Body Composition Analyser, Tanita, Japan). Habitual dietary intake was assessed using a written diary for 72 h, with diaries analysed for total energy intake and macronutrient composition of the diet using Nutritics software (Nutritics Ltd, Dublin, Ireland). At the first visit, participants also completed a food frequency questionnaire, which listed the quantity and frequency of anthocyanin-containing foods and drinks compiled from the Phenol Explorer database [23]. By multiplying the anthocyanin content of the portion size by the total consumption frequency of each food, daily anthocyanin intake was calculated (Table 1).

### Study 1 experimental design—acute supplementation

Study 1 required participants to undertake two experimental trials separated by a washout period of ≥ 7 days. 24 h before each experimental trial participants consumed a standardised diet (50% carbohydrate, 30% fat, 20% protein) that was otherwise matched to habitual energy intake. Participants were also instructed to abstain from vigorous exercise for 48 h and alcohol and caffeine for 24 h prior. On the morning of each experimental trial, participants attended the laboratory following an overnight fast (> 10 h) and first consumed a standardised high-carbohydrate breakfast (70% CHO, 10% protein, 20% fat, and equivalent to 25% of daily caloric intake) generally consisting of Weetabix with semi-skimmed milk, orange juice, an Upbeat protein drink and a banana. After they had consumed the breakfast they worked at a computer or sat quietly for 3 h. In a randomised, double-blind, crossover design, participants then ingested 2 capsules of NZBC extract (600 mg) or a visually-identical placebo, with water, 30 min prior to lunch. Each 300 mg NZBC capsule contained 105 mg of anthocyanins, consisting of 35–50% delphinidin-3-rutinoside, 5–20% delphinidin-3-glucoside, 30–45% cyanidin-3rutinoside, and 3–10% cyanidin-3-glucoside (CurraNZ™, Health Currancy Ltd., Surrey, UK). Each placebo capsule contained 300 mg microcrystalline cellulose. Following ingestion of either the NZBC extract or placebo, an indwelling cannula was placed into the antecubital vein of one arm and an initial blood sample was obtained. Thirty min following ingestion of NZBC extract or placebo, participants consumed a high-carbohydrate, high-fat liquid test meal consisting of 75 g maltodextrin (MyProtein™, The Hut Group, Cheshire, UK) and 50 g unsaturated fatty acids (Calogen, Nutricia, Amsterdam, NL). Blood samples were subsequently collected at 15 min intervals for the first hour and 30 min intervals for the remaining two hours. Once the testing procedure was completed the cannula was removed and participants were able to leave the laboratory.

### Study 2 experimental design—short-term supplementation

**Fig. 1:**
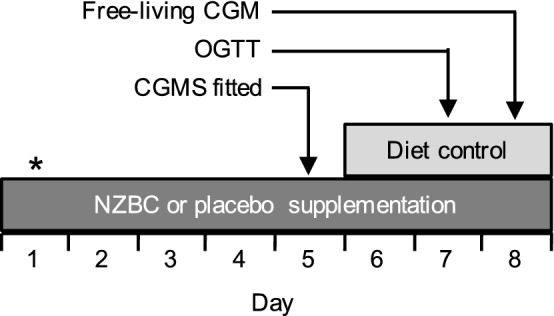
Schematic overview of experimental protocol for study 2. Participants undertook 8 days supplementation with NZBC extract (210 mg anthocyanins per day) or placebo, in a randomised, double-blind design. On day 1 participants provided a fasted blood sample*, and returned to the laboratory on day 5 to be fitted with a continuous glucose monitoring system (CGMS). A standardised diet was provided on days 6, 7 and 8 (50% carbohydrate, 30% fat, 20% protein), matched to each participant’s habitual energy intake. Participants underwent an oral glucose tolerance test (OGTT) on day 7, and 24 h glucose concentrations were collected under free-living conditions on day 8 using CGMS.

In a randomised, double-blinded, crossover design participants undertook 8 days supplementation with either NZBC extract (600 mg per day) or a visually-identical placebo. The supplement and placebo were identical to that used in study 1. One 300 mg capsule was ingested prior to breakfast, and one 300 mg capsule was ingested before dinner throughout the supplementation period. An overview of the experimental design for study 2 is provided in Fig. 1. On day 1 of each supplementation period a fasted blood sample was obtained from the antecubital vein of one arm. On day 5, participants were fitted with a continuous glucose monitoring system (CGMS) (described below), and provided with a standardised diet to be consumed on days 6, 7 and 8. On day 7, participants returned to the laboratory following an overnight fast (> 10 h) to undergo an oral glucose tolerance test (OGTT). Following collection of a fasted blood sample from an indwelling cannula placed in an antecubital vein, participants consumed 75 g maltodextrin (MyProtein™, The Hut Group, Cheshire, UK) diluted in 225 ml of water. Further blood samples were collected after 15, 30, 45, 60, 90 and 120 min, and collected into EDTA-containing vacutainers. Isotonic saline was used to keep the cannula patent every 15 min during the OGTT. On day 8, participants undertook their usual daily activities and the CGMS was used to examine interstitial glucose concentrations under free-living conditions. Each cross-over trial was separated by ≥ 15 days, which is based on a previous study that provided an anthocyanin dose greater than that used in the current study for 1 month and 15 days were required for antioxidant biomarkers to return to baseline levels (Alvarez-Suarez 2014).

#### Continuous glucose monitoring

A Dexcom G4 Platinum CGM probe (Dexcom, San Diego, CA, USA) was inserted subcutaneously into the lower abdominal region on day 5 of each supplementation period. This provided adequate time for “bedding in” and for the participants to become accustomed to using the CGMS. Participants were trained how to use the device and instructed to calibrate the device a minimum of four times daily using capillary blood samples. The monitor remained in place for the next 4 days, during which participants were provided with a standardised diet to consume that was matched to habitual energy intake but with a set macronutrient content (see Table 2 for overview of energy and macronutrient composition). On day 8, free-living glucose responses were assessed. On this day, participants were instructed to undertake their habitual daily activities, but consume their meals at pre-defined time points; 7–9 am breakfast, 12–2 pm lunch, and 5–7 pm evening meal. These times were chosen to ensure that there was a minimum 3 h postprandial period between meals.

**Table 2:** Macronutrient composition and intake during the free-living CGMS day (day 8)

	Macronutrient composition (% CHO/Fat/Protein)	Carbohydrate (g.kg body mass^−1^)	Fat (g.kg body mass^−1^)	Protein (g.kg body mass^−1^)
Breakfast	73%/13%/14%	6.3 ± 1.1	1.0 ± 0.2	1.2 ± 0.2
Lunch	54%/24%/22%	4.6 ± 0.8	1.8 ± 0.3	1.9 ± 0.3
Dinner	35%/45%/20%	4.0 ± 0.7	5.1 ± 0.9	1.9 ± 0.6
24 h	54%/26%/20%	5.0 ± 0.9	2.6 ± 0.5	1.7 ± 0.4

Values are means ± S.D. for carbohydrate, fat and protein

### Blood sample analysis

Across both studies, plasma samples for each time point were obtained following centrifugation (10 min at 1000 g at 4 °C) and stored at − 80 °C for subsequent analyses. Plasma glucose and triglyceride concentrations were determined spectrophotometrically using a semiautomatic analyser in combination with commercially available kits (Randox Laboratories, Antrim, UK). Plasma insulin, high-sensitivity interleukin-6 (hsIL-6), C-reactive protein (CRP) and hsTNF-ɑ concentrations were determined using commercially available ELISA kits (Invitrogen, Thermo Fisher Scientific, UK). For all assays the intra-assay coefficient of variation was ≤ 8.5% and the inter-assay coefficient of variation was ≤ 9.8%. The analytical sensitivity of the assays for insulin, CRP, hsIL-6 and hsTNF-ɑ was 0.17 µIU mL^−1^, < 10 pg mL^−1^, 0.03 pg mL^−1^, and 0.13 pg mL^−1^, respectively. Each sample was analysed in duplicate.

### Calculations and statistical analysis

Area under the curve (AUC) for plasma glucose, insulin and triglycerides was calculated using the conventional trapezoid rule. Insulin sensitivity was assessed using the homeostatic model assessment (HOMA) index and Matsuda [24] insulin sensitivity index. CGMS data were downloaded from the device using Dexcom Studio™ software (12.0.4.6) and first the glucose responses to each meal were investigated. In this regard, the 3 h postprandial period was evaluated for mean, peak and end glucose concentrations, and the area under the curve for the entire postprandial period was also calculated. All statistical analyses were performed using SPSS (v26.0, Chicago, IL, USA). Results are expressed as means ± S.D, and significance was set at the 0.05 level of confidence. For both study 1 and 2, time-dependent variables were assessed using a two-factor repeated-measures ANOVA, with the within-subject factors ‘condition’ (NZBC vs. placebo) and ‘time’. Significant main effects and interactions were assessed using Bonferroni adjustment post-hoc analysis. All other variables were investigated using a paired t-test. Both studies were powered to detect differences in glucose AUC between conditions (NZBC vs. placebo), with G*Power 3.1 software (G*Power Software Inc., Kiel, Germany) used to calculate the required sample size. A medium effect size (*f* = 0.30) was adopted and deemed to be physiologically-relevant, based on the data from two previous studies [7, 15], and used alongside an alpha of 0.05 and power of 0.80, to calculate the required sample size.

## Results

### Study 1—acute supplementation

**Fig. 2:**
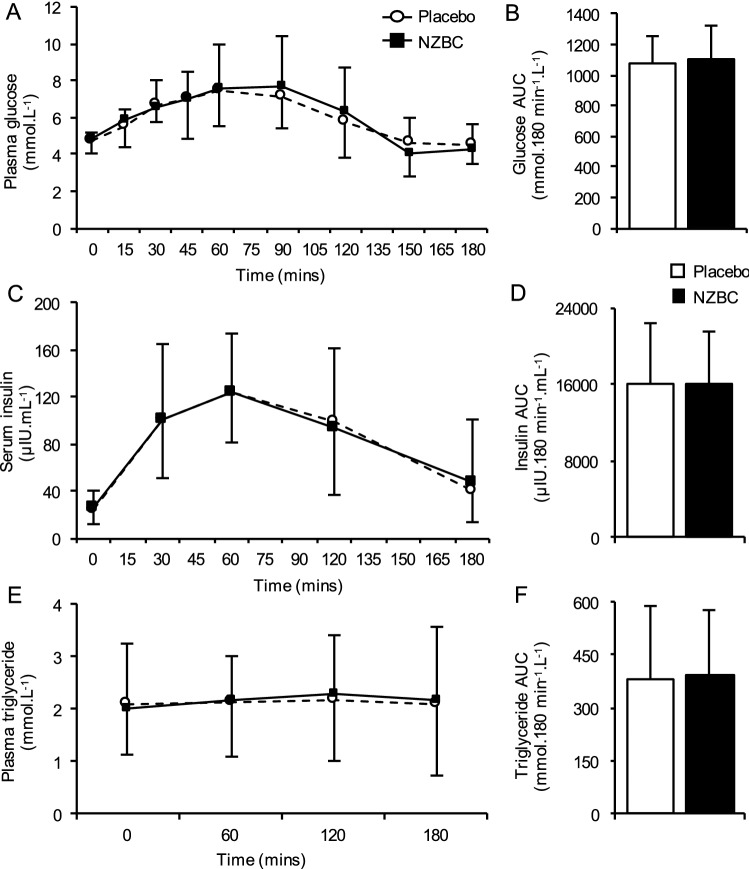
Postprandial responses to acute ingestion of NZBC extract or placebo. Participants ingested a single dose of NZBC extract (210 mg anthocyanins) or placebo, in a randomised, double-blinded design, 30 min prior to consuming a high-carbohydrate (75 g glucose), high-fat (50 g) liquid meal. 3 h postprandial plasma glucose (**a**), serum insulin (**c**), and plasma triglyceride concentrations (**e**), and the corresponding area under the curve (**b**, **d**, **f**, respectively). Values are presented as means ± S.D. (*n* = 12)

Plasma glucose, insulin and triglyceride responses to the carbohydrate-fat test drink are depicted in Fig. 2. Baseline plasma glucose, insulin and triglyceride concentrations were not different between conditions. In response to the carbohydrate-fat test drink, there were main time effects for plasma glucose and insulin concentrations (*P* = 0.02), although neither peak glucose nor insulin concentrations differed between conditions. In contrast, there was no main effect of time for plasma triglyceride concentrations in response to the carbohydrate-fat test drink (*P* = 0.21). Finally, AUC_glucose_, AUC_insulin_, or AUC_triglyceride_ were not different between conditions.

### Study 2—short-term supplementation

#### Glucose tolerance and insulin sensitivity (Fig. 3)

**Fig. 3:**
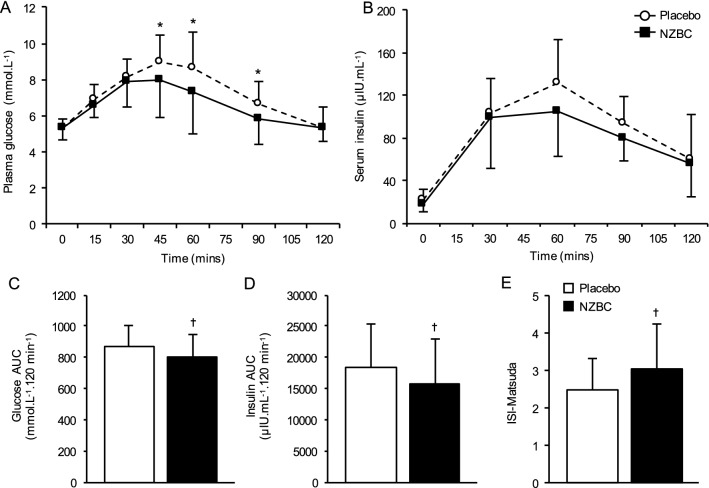
Effect of short-term supplementation with NZBC extract or placebo on glucose tolerance and insulin sensitivity. Concentration time-course responses of plasma glucose (a) and serum insulin (b) to an oral glucose tolerance test, and the corresponding area under the curves (c and d, respectively), following short-term (7 days) supplementation with NZBC extract (210 mg anthocyanins per day) or placebo. Whole-body insulin sensitivity was calculated using the Matsuda index [24] (e). Values are presented as means ± S.D. (*n* = 13). *P < 0.01 vs. corresponding placebo value (time × condition interaction; *P* = 0.048). ^†^*P* < 0.05 vs. placebo

Fasting plasma glucose and insulin concentrations were not different between conditions. There was a main time effect for both glucose (*P* < 0.001) and insulin (*P* = 0.002) during the OGTT, and mean plasma glucose concentrations were significantly lower following NZBC supplementation (*P* = 0.002). Furthermore, a significant time × condition interaction was observed for glucose (*P* = 0.048), with post-hoc analysis revealing significant reductions in plasma glucose concentrations following NZBC supplementation at 45 min (− 1.0 ± 0.9 mmol L^−1^; *P* = 0.003), 60 min (− 1.3 ± 1.0 mmol L^−1^; *P* = 0.001) and 90 min (− 0.8 ± 0.7 mmol L^−1^; P = 0.008) of the OGTT. Finally, both AUC_glucose_ (− 76 ± 48 mmol L^−1^.120 min^−1^; − 8%; *P* < 0.001) and AUC_insulin_ (− 2487 ± 2315 µIU mL^−1^.120 min^−1^; − 14%; *P* = 0.032) were reduced following NZBC supplementation compared to placebo. While HOMA index of insulin sensitivity tended to improve after NZBC supplementation (Placebo: 5.1 ± 2.5, NZBC: 4.4 ± 2.0; *P* = 0.053), whole-body insulin sensitivity assessed using the Matsuda insulin sensitivity was significantly increased (22%; *P* = 0.011) in response to NZBC supplementation compared to placebo.

#### Free-living glucose excursions and glycaemic variability

**Fig. 4:**
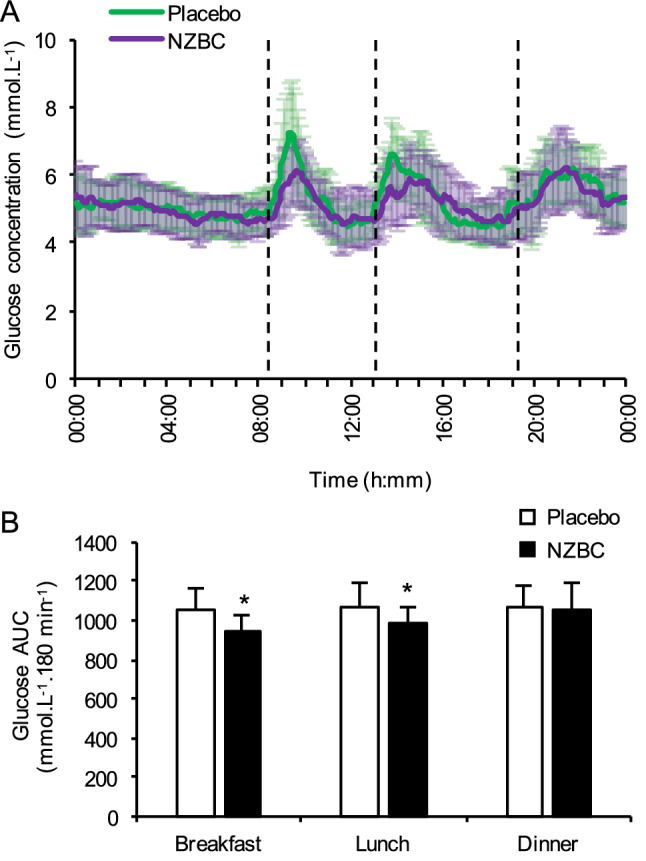
Daily free-living glucose concentrations and postprandial responses to short-term supplementation with NZBC extract or placebo. **a** Average glucose concentrations over time under standardised dietary, but otherwise free-living conditions, on the 8th day of supplementation with NZBC extract or placebo. The time-point at which the main meals were consumed is indicated by the dashed vertical lines. **b** Area under the curve for the 3 h postprandial period following each main meal, derived from the CGMS on day 8 of supplementation. Values are presented as means ± S.D. (*n* = 13). *P < 0.05 vs. placebo

The 3 h postprandial AUC_glucose_ response was 9% lower at breakfast (− 99 ± 110 mmol L^−1^.120 min^−1^; *P* = 0.01) and 8% lower at lunch on the free-living day (− 82 ± 105 mmol L^−1^.120 min^−1^ *P* = 0.021) following NZBC supplementation compared to placebo (Fig. 4). However, 3 h postprandial AUC_glucose_ response to dinner was not significantly different between conditions (Fig. 4). The mean glucose level at breakfast was significantly lower following NZBC supplementation (*P* = 0.03), tended to be lower at lunch (*P* = 0.059), but was not different at dinner (Table 3). Neither peak glucose levels, nor the final postprandial glucose levels following each meal during the free-living day, were different between conditions (Table 3).

**Table 3:** Key postprandial metrics from CGMS data

	Placebo	NZBC
Breakfast		
Mean glucose (mmol L^−1^)	5.77 ± 0.69	5.36 ± 0.43*
Peak glucose (mmol L^−1^)	7.46 ± 1.41	6.87 ± 0.73
End value (mmol L^−1^)	4.65 ± 0.59	4.69 ± 0.93
Lunch		
Mean glucose (mmol L^−1^)	5.84 ± 0.72	5.52 ± 0.40
Peak glucose (mmol L^−1^)	7.14 ± 1.15	6.69 ± 0.51
End value (mmol L^−1^)	5.09 ± 1.02	5.24 ± 0.68
Dinner		
Mean glucose (mmol L^−1^)	5.97 ± 0.55	5.75 ± 0.65
Peak glucose (mmol L^−1^)	6.97 ± 0.72	6.92 ± 0.92
End value (mmol L^−1^)	5.70 ± 0.81	5.83 ± 1.08

Values are means ± S.D

*P < 0.05 vs. placebo

#### Inflammatory markers (Table 4)

**Table 4:** Effect of NZBC extract on circulating markers of inflammation

	Placebo		NZBC	
	Pre	Day 7	Pre	Day 7
CRP (mg L^−1^)	1.71 ± 0.73	1.60 ± 0.82	1.69 ± 0.85	1.27 ± 0.63*
IL-6 (pg mL^−1^)	1.63 ± 0.86	1.76 ± 1.37	1.59 ± 0.74	1.70 ± 1.46
TNF-ɑ (pg mL^−1^)	1.49 ± 0.34	1.52 ± 0.26	1.56 ± 0.41	1.54 ± 0.42

Values are means ± S.D

*P < 0.05 vs. corresponding Pre value

Serum CRP concentrations were reduced in response to NZBC supplementation (*P* = 0.008), but not in response to placebo. In contrast, NZBC supplementation had no effect serum hsIL-6 or hsTNF-ɑ concentrations. All values were within the detection limits for each assay.

## Discussion

The key novel observations from the two studies described are: (1) acute ingestion of NZBC extract did not improve the postprandial glucose, insulin or triglyceride responses to a high-carbohydrate, high-fat meal in individuals with overweight or obesity; and (2) short-term (8 days) supplementation with NZBC extract improved free-living postprandial glucose responses and increased whole-body insulin sensitivity in individuals with overweight or obesity. Taken together, these findings suggest that repeated intake of anthocyanin-rich NZBC extract is required to induce a beneficial effect on insulin sensitivity and postprandial glucose excursions to ‘real world’ mixed-macronutrient meals.

A number of previous studies have reported a reduction in postprandial glucose and insulin excursions following a high-carbohydrate meal when the meal was preceded by, or combined with, anthocyanin-rich berries or berry-derived extracts [7–9, 12–14]. This effect is purported to be linked to the capacity for anthocyanins to (1) inhibit salivary and pancreatic ɑ-amylase and ɑ-glucosidase, thereby suppressing carbohydrate digestion, and (2) reduce the activity and/or expression of sodium-dependent glucose transporter-1 and glucose transporter-2 in the gut [25, 26], thereby reducing glucose absorption. Although these studies provide proof-of-concept evidence for the ability of anthocyanins to improve postprandial glucose excursions, they do not provide insight into the capacity for anthocyanins to modulate postprandial responses to meals consisting of a mixture of macronutrients. Therefore, in this study, we first sought to determine the effect of acute blackcurrant anthocyanin ingestion on postprandial responses to a mixed-macronutrient challenge representative of a westernised (i.e. high-carbohydrate, high-fat) meal. The first novel finding of the present study, and contrary to our hypothesis, was that acute intake of NZBC extract had no effect on postprandial glucose, insulin or triglyceride responses to a high-carbohydrate, high-fat liquid meal. By including fat in the test drink, which naturally delays glucose digestion and absorption [18], it is possible that any potential effect of NZBC extract to mediate postprandial glucose responses to the high-carbohydrate, high-fat challenge was masked by the fat component. A similar finding was reported by Edirisinghe et al. [9] who also failed to observe reduced postprandial glucose concentrations to a high-carbohydrate, moderate-fat meal following consumption of a beverage containing strawberry anthocyanins. The timing of the high-carbohydrate, high-fat challenge in our study should also be considered. In this context, the experimental meal was provided at lunch ≥ 3 h following a high-carbohydrate breakfast. This is important because postprandial glucose responses to a mixed-macronutrient meal are dampened when preceded by a meal several hours earlier [27, 28], termed the ‘second-meal phenomenon’. Thus, it is also possible that the preceding breakfast may have masked the effect of NZBC extract to suppress postprandial glucose responses to the high-carbohydrate, high-fat challenge.

Because there was no apparent benefit of acute NZBC intake on postprandial glucose responses, we next investigated whether repeated intake of NZBC extract was required to improve postprandial glucose responses to mixed-macronutrient meals. Accordingly, the second novel finding of this study is that repeated NZBC extract intake over 8 days reduced postprandial glucose responses to both breakfast and lunch meals in individuals with overweight/obesity. It was notable, though, that there was no improvement in the postprandial glucose response to dinner, although this could be, at least partly, due to the smaller proportion of carbohydrate in this meal (compared to breakfast and lunch). Nevertheless, by leveraging CGMS we were able to observe, for the first time, improved postprandial glucose responses under free-living conditions, albeit with standardised dietary control where participants were instructed to maintain their habitual daily activities. The translational nature of the CGMS data suggests, therefore, that NZBC extract could be a simple nutritional strategy to improve postprandial glucose responses in the ‘real world’.

Interestingly, the improved postprandial glucose responses to breakfast and lunch meals occurred in the absence of a change in peak postprandial glucose concentrations, and the 3 h postprandial (end) glucose concentrations were also similar between placebo and NZBC. This suggests that the overall effect of NZBC extract appears to be related to an increased rate of glucose clearance. To support this assertion, a significant decrease in plasma glucose and insulin concentrations was also observed during the OGTT following NZBC extract supplementation. The latter is important in the context of the OGTT, because it suggests that less insulin is required to clear the same quantity of glucose from the circulation. In line with this and for the first time, our data reveal that short-term NZBC intake improved whole-body insulin sensitivity in overweight and obese individuals. Improvements in insulin sensitivity have been reported following supplementation with anthocyanin-rich blueberry powder for 6 weeks in obese individuals, [11], but not freeze-dried blueberries for 6 months in individuals with metabolic syndrome [29]. Whether longer-term supplementation with NZBC intake also results in improved insulin sensitivity should be the focus of future work.

The overweight/obese phenotype is often associated with low-grade chronic inflammation, which is believed to promote insulin resistance in these individuals [3]. Several biomarkers of low-grade chronic inflammation were investigated, each of which has been reported to be positively associated with insulin resistance [3, 30]. In this regard, CRP, but not hsIL-6 or hsTNF-ɑ, was reduced in response to short-term supplementation with NZBC extract compared to placebo. This is the first study to report an effect of blackcurrant anthocyanins alone on CRP, although several studies have observed a reduction in CRP concentrations following supplementation with anthocyanins derived from a combination of blackcurrants and bilberries [31–33]. Notably though, these studies were ≥ 3 weeks in duration and used a dose of ≥ 300 mg anthocyanins per day. Therefore, our data provides new insight demonstrating that circulating CRP concentrations are sensitive to change to as little as 210 mg anthocyanins per day over a 7 day supplementation period. CRP is produced by the liver in response to inflammation [34], and directly impairs hepatic insulin sensitivity in rodents [35]. Furthermore, HOMA-IR, which reflects hepatic insulin resistance, tended to be reduced following 7 days intake of NZBC extract, in line with the results of a recent meta-analysis [36]. Thus, the reduction in circulating CRP concentrations provides evidence for the potential of NZBC extract to improve hepatic insulin sensitivity, and thereby contribute to the observed improvement in whole-body insulin sensitivity. Further studies are required to identify additional mechanisms underpinning the increase in whole-body insulin sensitivity in response to NZBC extract supplementation.

A strength of this work is the ability to demonstrate that improved postprandial glucose responses to mixed-macronutrient meals only occurred following short-term supplementation with NZBC extract. This observation was only possible because we examined the time-course response to NZBC intake, which in itself is a strength of the study. The bioavailability of anthocyanins is relatively low; only ~ 12% of ingested anthocyanins appear in the blood [37]. However, anthocyanin metabolites remain in the blood for up to 48 h following ingestion [38], and therefore repeated intake of NZBC will likely result in an accumulation of blackcurrant anthocyanin metabolites over time. Whether it is the anthocyanin metabolites, or the anthocyanins themselves, which underpins the improved insulin sensitivity and postprandial glucose handling remains to be determined. A further strength is the use of CGMS, which provides important novel insight into the effectiveness of NZBC extract to improve postprandial glucose handling under normal free-living conditions. We also standardized dietary intake during the CGMS period, which could be considered both a strength and a limitation of the study. We acknowledge that controlling dietary intake precludes conclusions to be drawn regarding the effect of NZBC intake on postprandial responses to meals habitually consumed by participants. However, as this was a proof-of-concept study, it is important to standardize dietary intake during the CGMS period to be able to compare postprandial glucose responses between conditions. We also acknowledge that trials are required to determine whether the effect of NZBC extract on insulin sensitivity persists over a longer-duration, and also to investigate whether the beneficial effects described here translate to more insulin resistant populations, such as T2D patients. In this regard, though, our study does reveal that NZBC extract can induce improvements in insulin sensitivity and postprandial glucose handling even in relatively metabolically healthy overweight and obese individuals.

In summary, we show for the first time that short-term, but not acute intake, of NZBC extract improves postprandial glucose handling and whole-body insulin sensitivity in individuals with overweight or obesity. Importantly, the beneficial effect of NZBC extract was observed under standardised dietary, but otherwise free-living conditions. We also report that NZBC extract reduced circulating CRP concentrations, highlighting that reduced hepatic inflammation may be one mechanism by which NZBC extract improves insulin sensitivity.
